# Prevalence and Prognostic Significance of Hyponatremia in Patients With Lung Cancer: Systematic Review and Meta-Analysis

**DOI:** 10.3389/fmed.2021.671951

**Published:** 2021-12-07

**Authors:** Eszter Bartalis, Marin Gergics, Benedek Tinusz, Mária Földi, Szabolcs Kiss, Dávid Németh, Margit Solymár, Zsolt Szakács, Péter Hegyi, Emese Mezösi, László Bajnok

**Affiliations:** ^1^Medical School, Institute for Translational Medicine, University of Pécs, Pécs, Hungary; ^2^University of Medicine, Pharmacy, Science and Technology of Târgu Mureş, Târgu Mureş, Romania; ^3^First Department of Medicine, Medical School, University of Pécs, Pécs, Hungary; ^4^Szentágothai Research Centre, University of Pécs, Pécs, Hungary; ^5^Doctoral School of Clinical Medicine, University of Szeged, Szeged, Hungary

**Keywords:** lung cancer, NSCLC, SCLC, hyponatremia, SIADH

## Abstract

**Background:** The prevalence of hyponatremia is highly variable among patients with lung cancer. However, its prevalence and prognostic significance in subgroups of patients with lung cancer have not yet been evaluated in a meta-analysis.

**Methods:** We have registered our meta-analysis and review protocol to the PROSPERO International Prospective Register of Systematic Reviews, with the following registration number: CRD42020167013. A systematic search was done in the following sources: MEDLINE, Embase, CENTRAL, Web of Science, ClinicalTrials.gov, a WHO Global Health Library.

**Results:** We identified a total of 8,962 potentially eligible studies, and we included 31 articles in our evaluation. The prevalence of hyponatremia in patients with lung cancer varied between 3 and 94.8% with an average of 25% without any significant differences between the following subgroups: histotype, gender, age, Eastern Cooperative Oncology Group (ECOG) state, and the extent of disease. The overall survival (OS) was significantly lower in hyponatremic compared to normonatremic patients at 10 months [RR.59 (95% CI.47–0.74), *p* < 0.001] and at 20 months [RR.44 (95% CI.33–0.59), *p* < 0.001], with worse survival rates in non-small cell lung cancer (NSCLC) [RR.27 (95% CI.12–0.44), *p* < 0.001] than in small cell lung cancer (SCLC) [RR.42 (95% CI.27–0.57), *p* < 0.001]. If hyponatremia was corrected, OS at 10 months was significantly higher than in the uncorrected hyponatremia group [RR 1.83 (95% CI 1.37–2.44), *p* < 0.001], but, at 20 months, no statistically significant difference could be found between these subgroups [RR 2.65 (95% CI.94–7.50), *p* = 0.067].

**Conclusions:** Patients with lung cancer diagnosed with hyponatremia, especially patients with NSCLC, seem to have significantly lower survival rates than normonatremic patients. If hyponatremia remains uncorrected, the mortality rates might be even higher.

## Background

Lung cancer is one of the most frequent malignancies worldwide in both sexes, and 75% of patients are diagnosed at an advanced stage. According to the reports of the WHO in 2018, it is the leading cause of mortality among cancers (1). Moreover, based on the WHO's Global Cancer Observatory's report, the incidence of lung cancer is predicted to grow by 72.5% by 2040 (2).

The reported prevalence values of hyponatremia (<135 mmol/L) among patients with lung cancer are highly discordant in the literature, varying between 3.7 and 75% (3–8). Although inappropriate antidiuretic hormone secretion (SIADH) is the most common underlying cause (4, 5), the prevalence of SIADH seems to be lower among patients with lung cancer compared to hyponatremia, varying from 9.1 to 39% in small cell lung cancer (SCLC) (6, 9–11) and 7–4% in non-small cell lung cancer (NSCLC) (6, 12). SIADH was diagnosed even less frequently in the general cancer population (4, 13, 14). This discrepancy may be partially explained by insufficient diagnostic work-up and the presence of other comorbidities that also affect the sodium level (15–17).

Based on literature reports, hyponatremia and SIADH appear to be important negative prognostic factors of mortality in numerous medical conditions, including lung cancer (4, 9, 12, 18–22), but not every study reached this conclusion (6, 10). However, a recent meta-analysis of 15 studies confirmed the prognostic significance of serum sodium levels by demonstrating that the correction of hyponatremia is associated with a reduced risk of mortality in hospitalized patients with oncology (23).

Following a preliminary search of the literature, we found discordant results regarding the prevalence and prognostic significance of hyponatremia in patients with lung cancer, and the findings considerably differ from the studies and reviews cited above. This study aims to make a reappraisal of the occurrence and prognostic impact of pre and posttreatment (oncological, symptomatic, and supportive) hyponatremia, specifically in patients with lung cancer.

## Methods

### Protocol and Registration

We report this systematic review and meta-analysis according to the preferred reporting items for systematic reviews and meta-analyses (PRISMA, 2009) Statement (24) described in Supplementary File 1. We have registered our meta-analysis and review protocol to the PROSPERO International Prospective Register of Systematic Reviews, with the following registration number: CRD42020167013. There were no deviations from the study protocol.

### Search Strategy

The first step was to create a PECO (patient-exposure/prognostic factor-comparison-outcome): “P,” patients with lung cancer; “E,” hyponatremia at the time of the diagnosis/before treatment; “C,” normonatremia at the time of the diagnosis/before treatment; “O,” overall survival time and the rate at 10 and 20 months. Then, we created a search key based on our PECO, which included search terms related to the group of the patients and the prognostic factor: (*lung cancer OR SCLC OR NSCLC OR carcinoid) AND (SIADH OR the sodium level OR the Na level OR hyponatremia OR hyponatremia OR syndrome of inappropriate ADH OR antidiuretic hormone OR hypotonicity*).

We searched the following sources: MEDLINE (*via* PubMed), Embase, Cochrane Central Register of Controlled Trials (CENTRAL), Web of Science, ClinicalTrials.gov, and WHO Global Health Library. The “human” filter was applied in the case of MEDLINE, Embase, and the WHO Global Health Library. The “trials” and “completed” filters were utilized when searching in the Cochrane Library or ClinicalTrials.gov, respectively. We did not apply any language or date restriction to our search. We also performed a search on the bibliography of the eligible articles. The date of the search was 12.03.2019. Then, we updated our systematic search with the same search strategy used before on 05.07.2021.

### Eligibility and Exclusion Criteria

Studies qualified our systematic review after fulfilling the following requirements: (1) observational or interventional cohort studies, case-control studies, randomized-controlled trials, and case series. Besides full-text articles, conference abstracts were also eligible for inclusion; (2) trials enrolling adult patients (18 years or above) with lung cancer with available information of characteristics of patients with lung cancer (number of patients, age, sex, histological type of cancer, extent of disease, and performance status) and pretreatment serum sodium levels; (3) trials reporting data on overall survival (OS) time and the rate at 10 and 20 months, or with available information about multivariate analysis of the prognostic significance of hyponatremia.

The exclusion criteria were the following: (1) case studies, case reports, reviews, comments, and letters; (2) studies investigating other lung pathologies or solid cancers; (3) lack of data on serum sodium levels before treatment; (4) papers not reporting outcomes mentioned before.

### Study Selection and Data Extraction

After the initial search, all records from each database were imported into a reference management program (EndNote X7, Clarivate Analytics, Philadelphia, PA, USA). This software was used to remove duplicates. After the removal of duplicates, the authors screened the remaining articles against the predefined eligibility criteria first by title, abstract, and then full text. Two researchers conducted each step independently, and any disagreements were resolved by consensus.

Numerical data were extracted by two independent investigators, any disagreements were resolved by consensus, and data were manually entered on an Excel 2018 sheet (Office 365, Microsoft, Redmond, WA, USA). These were collected on the first author, year of publication, study design, geographical location, number of patients, and basic demographics (age and sex ratio), in each group. Additional information on the histological type of the tumor, disease extent, Eastern Cooperative Oncology Group (ECOG) Performance status, the grade of hyponatremia, and the length of follow-up was also obtained. We also collected information about hyponatremia improvement that was achieved by oncologic, symptomatic, and/or supportive treatment, including at least two cycles of chemotherapy, sodium supplementation, or fluid restriction.

#### Outcomes

Finally, data were collected on the primary and secondary outcomes of interest, such as the prevalence of hyponatremia, median OS time, and the OS rate at 10 and 20 months of patients with normonatremia and hyponatremia and results of multivariate analyses of lung cancer patients with hyponatremia.

### Risk of Bias Assessment

We used the Quality in Prognostic Studies (QUIPS) tool (25) for assessing the risk of bias, which is described in Supplementary File 2. Any disagreements between the two independent researchers were solved by consensus.

### Statistical Analysis

The effect measure of dichotomous variables was reported for each outcome as the relative risk (RR) with the related 95% CI. All tests were two-sided, and *p* < 0.05 was considered statistically significant (except for heterogeneity, for which a *p* < 0.10 was considered significant). The random-effects model was used because of the minor differences between individual studies.

Heterogeneity was tested by performing both Cochran's Q test and calculating Higgins' I^∧^2 indicator. *p* < 0.10 was considered suggestive of significant heterogeneity. The I2 index corresponds to the percentage of the total variability across studies, which is due to heterogeneity. Based on the Cochrane Handbook for Systematic Reviews of Interventions (26), a rough classification of its value is the following: low (0–40%), moderate (30–60%), substantial (50–90%), and considerable (75–100%). All the statistical analyses were performed using Stata IC (version 15.1).

## Results

**Figure 1:**
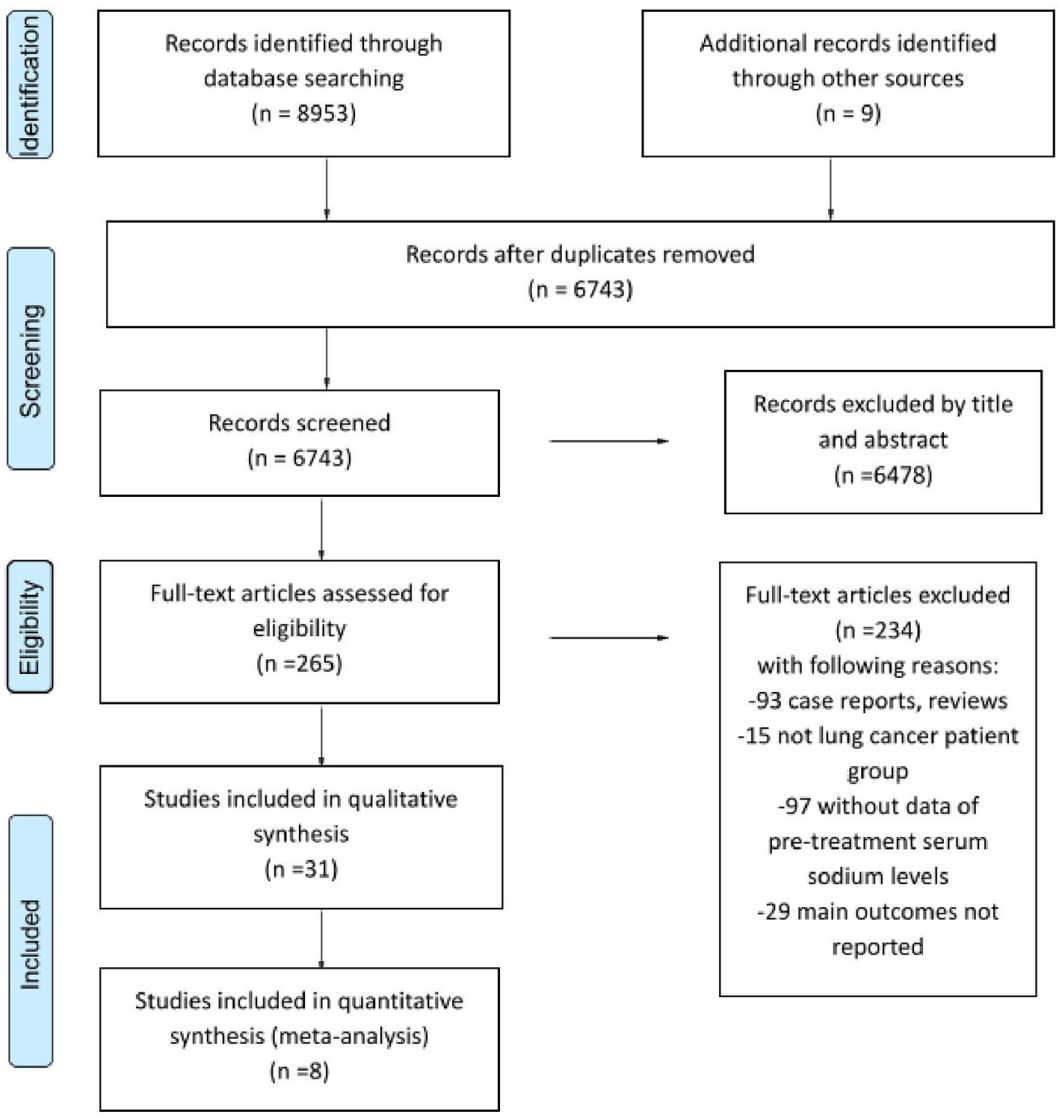
A preferred reporting items for systematic reviews and meta-analyses (PRISMA) flow diagram representing the process of study search and selection.

The selection process is summarized in Figure 1. We identified a total of 8,962 potentially eligible studies in the literature, 1,858 articles in MEDLINE, 5,201 articles in Embase, 315 records in CENTRAL, 1,440 articles in Web of Science, 33 in ClinicalTrials.gov, and 115 in WHO Global Health Library. After screening by title, abstract, and full text, we chose 31 studies for our evaluation based on our eligibility criteria. Since not all studies reported on every parameter, we could include only a limited number of studies in each sub-analysis.

### Systematic Review

The characteristics of the studies are summarized in a table included in Table 1. All selected reports were published in peer-reviewed journals, including four prospective and 27 retrospective cohort or case-control studies. The number of participants in the trials varied from 40 to 1,083 patients. Every selected article reported demographic information of the investigated population and data on the serum sodium levels at admission or before starting oncological treatment. In all of the 31 articles selected, we analyzed the prevalence of hyponatremia. However, the analysis of the prognostic significance was performed only in 26 studies due to the lack of data in some articles. Furthermore, only 15 studies reported on median OS times, and the OS rates at 10 and 20 months were available in even fewer studies (*n* = 8).

**Table 1:** Characteristics of the included studies in the systematic review.

**Study**	**Country**	**Study design**	**Nr. of patients**	**Histotype**	**Follow-up time**	**Male N (%)**	**Age cut-off (years)**	**Older** **N (%)**	**Na level cut-off (mmol/L)**	**Hyponatremia *N* (%)**
Yang et al. (27)	China	Retrospective	320	SCLC	40 weeks	219 (68.4%)	60	222 (69.4%)	135	*149 (46.6%)*
Osterlind et al. (28)	Denmark	Retrospective	815	SCLC	18–54 weeks	584 (71.7%)	60	428 (52.5%)	136	*204 (25.0%)*
Zarzecka et al. (29)	Poland	Retrospective	290	NSCLC	NR	205 (70.7%)	NR	NR	135	*47 (16.2%)*
Hermes et al. (30)	Germany	Retrospective	395	SCLC	NR	240 (60.8%)	60	260 (65.8%)	135	*75 (19.0%)*
Wang et al. (31)	China	Retrospective	631	SCLC	NR	475 (75.3%)	65	91 (14.4%)	135	*66 (10.5%)*
Berardi et al. (32)	Italy	Retrospective	433	NSCLC	NR	299 (69.1%)	NR	NR	135	*69 (15.9%)*
Sengupta et al. (33)	India	Retrospective	116	NSCLC +SCLC	NR	83 (71.6%)	NR	NR	135	*45 (45.5%)* *7 (41.2%)*
Kobayashi et al. (34)	Japan	Retrospective	386	NSCLC	41.2 months	259 (67.1%)	NR	NR	139	*123 (31.9%)*
Fucá et al. (35)	Italy	Prospective	197	NSCLC	25.7 months	120 (60.9%)	65	113 (57.4%)	135	*26 (13.2%)*
Alamoudi (36)	Saudi Arabia	Prospective	114	SCLC +NSCLC	NR	89 (78.1%)	NR	NR	130	*64 (56.1%)*
Hansen et al. (37)	Denmark	Retrospective	453	SCLC	60 months	243 (53.6%)	60	323 (71.3%)	135	*198 (44%)*
Svaton et al. (38)	Czech Republic	Retrospective	544	NSCLC	84 months	343 (63.1%)	65	242 (44.5%)	136	*117 (21.5%)*
Allan et al. (39)	U.K.	Retrospective	411	SCLC	NR	NR	65	120 (29.2%)	135	*111 (27.0%)*
Li et al. (40)	China	Retrospective	1083	NSCLC	40.84 months	757 (69.9%)	65	295 (27.2%)	141,9	*165 (15.2%)*
Osterlind et al. (41)	Denmark	Retrospective	874	SCLC	18 months	631 (72.2%)	60	450 (51.5%)	136	*213 (24.4%)*
Doshi et al. (42)	India	Retrospective	257	NSCLC	NR	180 (70.0%)	NR	NR	136	*120 (46.7%)*
Johnson et al. (43)	U.S.A.	Prospective	50	SCLC +NSCLC	NR	33 (66.0%)	NR	NR	130	*10 (32.3%)* *1 (5.3%)*
Sagman et al. (44)	Canada	Retrospective	614	SCLC	NR	436 (71.0%)	70	88 (14.3%)	135	*142 (23.1%)*
Jacot et al. (45)	France	Retrospective	301	NSCLC	20.8 months	242 (80.4%)	NR	NR	NR	*24 (8.0%)*
Hong et al. (46)	China	Retrospective	999	SCLC	10.6 months	692 (69.3%)	60	387 (38.7%)	135	*163 (29.5%)*
Maestu et al. (47)	Spain	Retrospective	341	SCLC	NR	336 (98.5%)	65	88 (25.8%)	135	*10 (3%)*
Kawahara et al. (48)	Japan	Retrospective	286	SCLC	NR	233 (81.5%)	66	113 (39.5%)	136	*40 (14.0%)*
Cerny et al. (49)	U.K.	Retrospective	407	SCLC	NR	262 (64.4%)	NR	NR	132	*53 (13.0%)*
Ma et al. (50)	China	Retrospective	158	SCLC	NR	135 (85.4%)	60	61 (38.6%)	135	*67 (42.4%)*
Umemura et al. (51)	Japan	Retrospective	163	SCLC	36 months	129 (79.1%)	70	69 (42.3%)	135	*22 (13.5%)*
Jacot et al. (52)	France	Retrospective	231	NSCLC	35 months	194 (84,0%)	60	104 (45%)	132	*219 (94.8%)*
Rechnitzer et al. (53)	Germany	Prospective	229	NSCLC	42 months	185 (80.8%)	63	69 (30.1%)	137.5	*59 (25,8%)*
Bose et al. (54)	India	Retrospective	40	NSCLC	6 months	38 (95%)	NR	NR	125	*8 (20%)*
Rinaldi et al. (55)	Italy and UK	Retrospective	647	NSCLC	NR	440 (68%)	65	NR	135	*105 (16.2%)*
Chan et al. (56)	Ireland	Retrospective	624	LC	NR	370 (59.3%)	NR	NR	135	*197 (31.6%)*
Huang et al. (57)	China	Retrospective	358	SCLC	NR	286 (79.9%)	70	49 (13.7%)	137	*54 (15.1%)*

*SCLC, Small cell lung cancer; NSCLC, Non-small cell lung cancer; RCT, Randomized control trial; U.S.A, United States of America; U.K., United Kingdom; NR, Not reported*.

The main clinical characteristics of patients in individual articles are shown in Tables 1, 2). Out of the 12,767 patients included, 57.8% (*N* = 7,379) were diagnosed with SCLC, 41.2% (*N* = 5,255) with NSCLC, 65% (8,298) were men and 27.3% were women. The reported median age ranged from 49 to 68 years. With a cut-off value of 60 years (eight studies), 52.6% of the studied population was older than 60 years, while, with a cut-off value of 65 years (seven studies), 70.4% was younger. In total, 2,973 patients had hyponatremia at admission or before starting oncological treatment. The applied cut-off values varied between 132 and 139 mmol/L across studies. Hyponatremia was diagnosed in 1,499 of 6,792 patients (22.1%) using a 135 mmol/L cut-off value in most of the included articles (16 studies), while, in five studies, where the cut-off value was 136 mmol/L, the number of patients with hyponatremia was 694 out of 2,776 patients (25%). In most cases, the oncological treatment for SCLC consisted of platinum-based preparations and etoposide, and, for NSCLC, were platinum-based preparations and/or surgery (Table 2).

**Table 2:** Different oncological treatment protocols in the included studies.

**Studies**	**Study interval**		**Histotype**	**Number of patients**	**Oncological treatment protocols**
Yang et al. (27)	2006	2012	SCLC	320	PB+E
Osterlind et al. (28)	1973	1981	SCLC	815	L+C+M, L+C+M+V, L+C+M+V+D+E alternating, L+C+E+V +/- radiotherapy
Zarzecka et al. (29)	2010	2012	NSCLC	290	NSCLC – PB and third generation drugs, in SCLC with PB+E
Hermes et al. (30)	2004	2008	SCLC	395	PB+E
Wang et al. (31)	2006	2013	SCLC	631	PB+E
Berardi et al. (32)	2006	2015	NSCLC	433	PB, Non-PB, EGFR- TKI
Sengupta et al. (33)	2011	2012	NSCLC	116	unknown
Kobayashi et al. (34)	2000	2009	NSCLC	386	resection+ postoperative adjuvant treatment
Fucá et al. (35)	2013	2018	NSCLC	197	PD-1/PDL-1 inhibitors
Alamoudi (36)	2004	2008	LC	114	unkown
Hansen et al. (37)	1995	2005	SCLC	453	PB+E
Svaton et al. (38)	2006	2013	NSCLC	544	erlotinib
Allan et al. (39)	1982	1988	SCLC	411	M+CCVVP+/-D,+/-radiotherapy and PCI or VI+E
Li et al. (40)	2007	2014	NSCLC	1083	resection
Osterlind et al. (41)	1973	1981	SCLC	874	L+C+M,L+C+M+V, L+C+M+V+D+E, +/- radiotherapy
Doshi et al. (42)	2010	2014	NSCLC	257	PB+PM
Johnson et al. (43)	1989	1992	LC	50	PB+E
Sagman et al. (44)	1976	1986	SCLC	614	Different protocols: C+D+V, L+P+M, PB+E
Jacot et al. (45)	2003	2006	NSCLC	301	surgery, PB, second line, palliative
Hong et al. (46)	2000	2009	SCLC	999	chemotherapy(etoposide)+/-radiotherapy+/-surgery
Maestu et al. (47)	1981	1993	SCLC	341	different protocols:C+D+V, PB and others+/- radio
Kawahara et al. (48)	1985	1988	SCLC	286	different protocols: C+D+V, P+E, or C+D+V+P+E
Cerny et al. (49)	1979	1985	SCLC	407	different protocols:C+M+E+/-radiotherapy
Ma et al. (50)	2000	2007	SCLC	158	unknown
Umemura et al. (51)	1981	2001	SCLC	163	PB or alternating chemotherapy
Jacot et al. (52)	1992	1998	NSCLC	231	radiotherapy, chemotherapy, surgery and their combinations
Rechnitzer et al. (53)	1987	1990	NSCLC	229	chemotherapy+radiotherapy
Bose et al. (54)	2008	2008	NSCLC	40	PB+PC for NSCLC, PB+E for SCLC
Rinaldi et al. (55)	2006	2017	NSCLC	647	PB or EGFR-TKI
Chan et al. (56)	2011	2016	LC	624	unknown
Huang et al. (57)	2011	2018	SCLC	358	PB+ E and others

*SCLC, small cell lung cancer, NSCLC, Non-small cell lung cancer, LC, every lung cancer histotype, PB, Platinum-based preparations, PC, paclitaxel, PM, pemetrexed, L, lomustine, C, cyclophosphamide, M, methotrexate, V, vincristine, D, doxorubicine, E, etoposide, P, procarbazine, VI, vindesine, PCI, prophilactic cranial irradiation, EGFR, epidermal growth factor receptor, TKI, tyrosine kinase inhibitors*.

The results of various outcomes among the selected studies are shown in Table 3. The prognostic significance of hyponatremia was evaluated in 26 studies and was found to be an independent prognostic factor in 15 studies, whereas, in eight studies, it was not.

**Table 3A:** The outcomes in lung cancer patients among the included studies.

**Study**	**Median OS times**		***p*-value**	**Survival rates, mortality**		***p*-value**	**Multivariate analysis**			
	**NN patients**	**HN patients**		**NN patients**	**HN patients**			**HR**	**95% CI**	***p*-value**
Yang et al. (27)	1.1 ± 0.42 year	0.83 ± 0.35 year	<0.05	62.26% 1-year SR 1.89% 5-year SR	30.36% 1-year SR 0% 5-year SR	0.045 0.15	NR	-	-	-
Osterlind et al. (28)	NR	NR	-	NR	NR	-	NR	-	-	-
Zarzecka et al. (29)	NR	NR	-	7.8% mortality	28.7% mortality	0.0001	NR	-	-	-
Hermes et al. (30)	13 months	9 months	<0.001	NR	NR	-	I	-	-	0.025 LD 0.038 ED
Wang et al. (31)	14.5 months	11.4 months	<0.001	NR	NR	-	I	1.82	1.34–2.47	0.007
Berardi et al. (32)	15.5 months	8.78 months	<0.001	57.06% SR	33.33% SR	0.19	I	1.59	1.14–2.21	0.006
Sengupta et al. (33)	NR	NR	-	NR	NR	-	NI	-	-	NS
Kobayashi et al. (34)	NR	NR	-	74.8% 5-year SR	59.7% 5-year SR	0.002	I	1.53	1.01–2.32	0.047
Fucá et al. (35)	11.6 months	2.8 months	<0.001	39.76% SR at 10 months	15.38% SR at 10 months	-	I	3.11	1.91–5.05	<0.001
Alamoudi (36)	NR	NR	-	NR	NR	-	NR	-	-	-
Hansen et al. (37)	11.2 months	7.1 months	0.0001	57.25% SR at 10 months	27.27% SR at 10 months	-	I	1.6	1.27–2.01	<0.001
Svaton et al. (38)	10.9 months	4.6 months	<0.001	54.33% SR at 10 months	21.36% SR at 10 months	<0.001	I	1.87	1.47–2.39	<0.001

*OS, overall survival; NN, normonatremic; HN, hyponatremic; NR, Not reported; S-significant; NS, not significant; I, Independent factor; N.I., Not independent factor; HR, hazard ratio; RR, risk ratio; SR, Survival rate; CI, confidence interval; LD, Limited Disease; ED, Extensive Disease*.

**Table 3B:** The outcomes in lung cancer patients among the included studies.

**Study**	**Median OS times**		***p*-value**	**Survival rates, mortality**		***p*-value**	**Outcome in multivariate analysis**			
	**NN patients**	**HN patients**		**NN patients**	**HN patients**			**HR**	**95% CI**	***p*-value**
Allan et al. (39)	7 months	7 months	0.06	NR	NR	-	NI	-	-	NS
Li et al. (40)	NR	NR	-	79.6% SR at 25 months	73.83% SR at 25 months	-	NR	-	-	-
Osterlind et al. (41)	40 weeks	34 weeks	-	NR	NR	-	I	-	-	<0.05
Doshi et al. (42)	16 months	11 months	<0.05	61.31% SR at 10 months	54.16% SR at 10 months	-	I	2.07	1.11–3.84	<0.05
Johnson et al. (43)	NR	NR	-	NR	NR	-	NI	-	-	NS
Sagman et al. (44)	45 weeks	42 weeks	0.006	NR	NR	-	NI	-	-	NS
Jacot et al. (45)	18.7 months	4.1 months	<0.0001	NR	NR	-	I	1.99	1.04–3.77	S
Hong et al. (46)	11.7 months	10 months	0.039	59.48% SR at 10 months	49.69% SR at 10 months	-	NI	-	-	NS
Maestu et al. (47)	NR	NR	-	NR	NR	-	NI	-	-	NS
Kawahara et al. (48)	11.4 months	9.1 months	0.0072	NR	NR	-	NI	-	-	NS
Cerny et al. (49)	NR	NR	-	59.48% SR at 10 months	34% SR at 10 months	-	I	-	-	0.0009
Ma et al. (50)	14.1 months	7.6 months	<0.001	81.31% SR at 10 months	32.83% SR at 10 months	-	NR	-	-	-
Umemura et al. (51)	10.6 months	10 months	0.6653	50% SR at 10 months	51.77% SR at 10 months	NS	NI	-	-	NS
Jacot et al. (52)	7.5 months	3.85 months	0.0141	NR	NR	-	I	2.99	1.17–7.62	0.022
Rechnitzer et al. (53)	6.7 months	3 months	<0.001	NR	NR	-	I	-	-	<0.001
Bose et al. (54)	NR	NR	<0.03	NR	NR	-	NR	-	-	-
Rinaldi et al. (55)	15.3 months	10.3 months	0.003	NR	NR	-	I	1.29	1.03–1.54	0.047
Chan et al. (56)	NR	NR	-	NR	NR	-	NR	-	-	-
Huang et al. (57)	14.5 months	11 months	0.008	NR	NR	-	I	1.49	1.04–2.13	0.03

*OS, overall survival; NN, normonatremic; HN, hyponatremic; NR, Not reported; S-significant; NS, not significant; I, Independent factor; NI, Not independent factor; HR, hazard ratio; RR, risk ratio; SR, Survival rate; CI, confidence interval*.

After evaluating the risk of bias in each study using the QUIPS tool, the included reports had a low, high, and most medium risk of bias (Table 4).

**Table 4:** Risk of bias assessment.

	**Study participation**	**Study attrition**	**Prognostic factor measurement**	**Outcome measurement**	**Study confounding**	**Statistical analysis and reporting**	**Overall risk of bias**	**Included in quantitative synthesis**
Yang et al. (27)	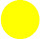	n.a.	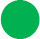	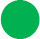	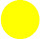	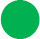	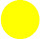	yes
Osterlind et al. (28)	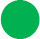	n.a.	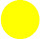	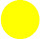	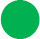	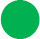	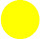	no
Zarzecka et al. (29)	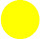	n.a.	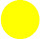	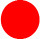	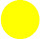	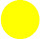	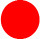	no
Hermes et al. (30)	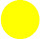	n.a.	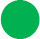	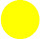	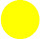	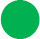	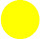	no
Wang et al. (31)	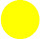	n.a.	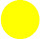	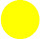	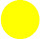	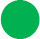	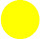	no
Berardi et al. (32)	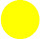	n.a.	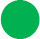	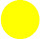	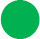	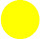	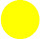	yes
Sengupta et al. (33)	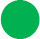	n.a.	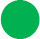	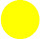	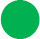	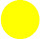	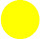	no
Kobayashi et al. (34)	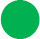	n.a.	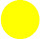	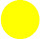	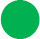	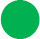	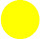	no
Fucá et al. (35)	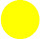	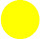	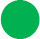	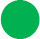	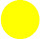	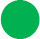	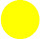	yes
Alamoudi (36)	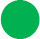	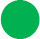	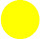	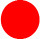	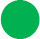	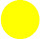	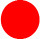	no
Hansen et al. (37)	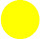	n.a.	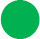	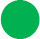	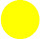	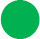	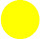	yes
Svaton et al. (38)	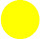	n.a.	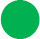	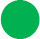	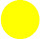	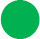	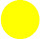	yes
Allan et al. (39)	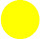	n.a.	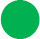	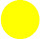	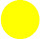	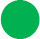	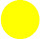	no
Li et al. (40)	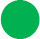	n.a.	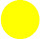	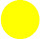	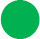	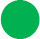	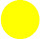	no
Osterlind et al. (41)	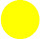	n.a.	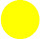	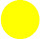	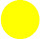	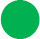	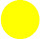	no
Doshi et al. (42)	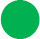	n.a.	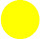	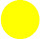	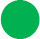	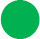	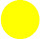	no
Johnson et al. (43)	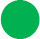	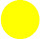	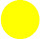	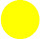	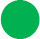	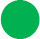	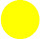	no
Jacot et al. (45)	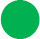	n.a.	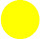	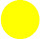	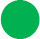	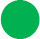	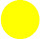	no
Umemura et al. (51)	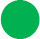	n.a.	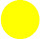	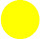	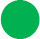	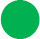	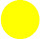	no
Sagman et al. (44)	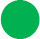	n.a.	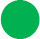	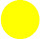	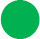	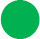	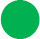	no
Hong et al. (46)	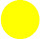	n.a.	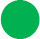	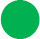	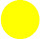	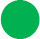	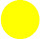	yes
Maestu et al. (47)	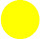	n.a.	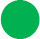	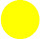	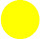	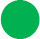	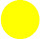	no
Kawahara et al. (48)	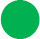	n.a.	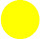	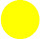	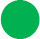	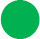	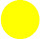	no
Cerny et al. (49)	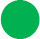	n.a.	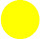	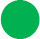	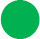	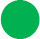	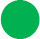	yes
Ma et al. (50)	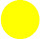	n.a.	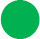	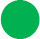	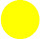	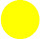	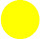	yes
Jacot et al. (52)	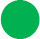	n.a.	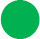	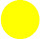	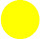	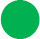	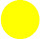	no
Rechnitzer et al. (53)	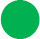	n.a.	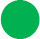	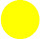	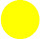	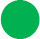	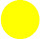	no
Bose et al. (54)	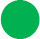	n.a.	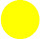	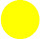	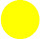	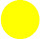	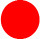	no
Rinaldi et al. (55)	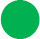	n.a.	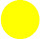	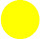	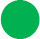	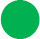	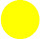	no
Chan et al. (56)	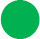	n.a.	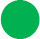	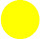	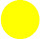	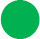	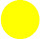	no
Huang et al. (57)	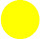	n.a.	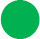	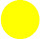	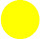	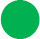	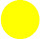	no
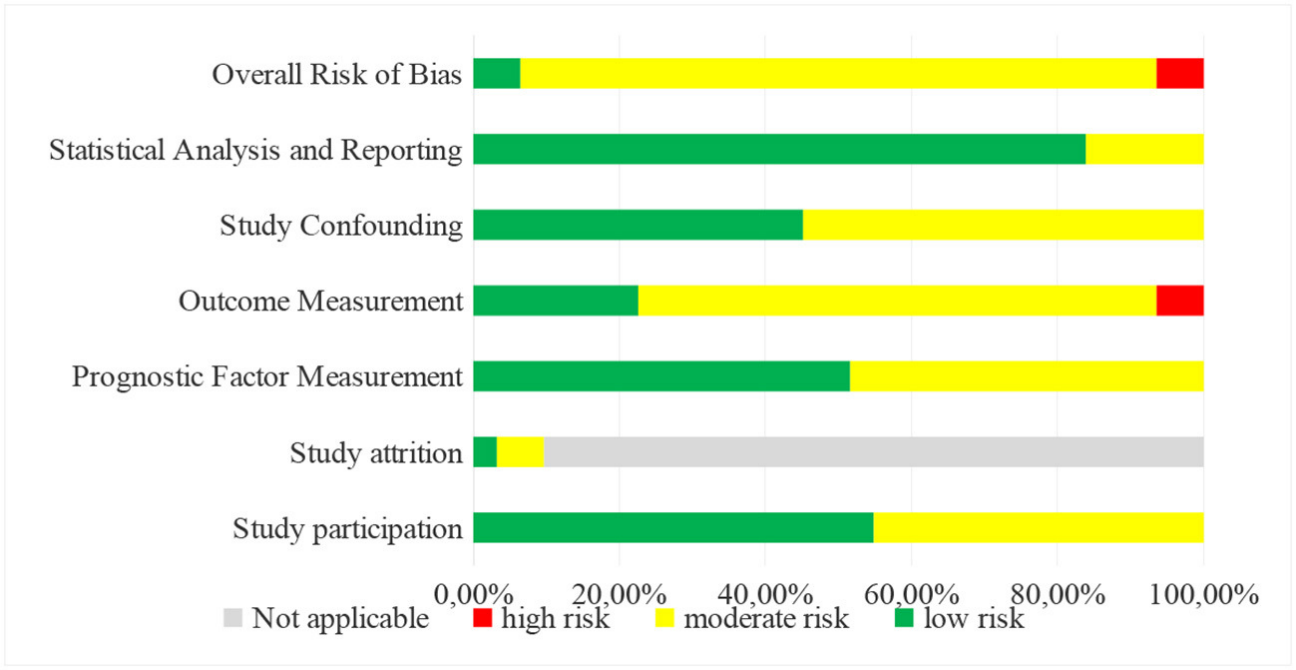								
[CHART]								

### Meta-Analysis

The summary of the results of the statistical analysis is presented in Table 5.

**Table 5:** Results of the statistical analyses.

**Prevalence of hyponatremia**					
**Subgroups**	**RR**	**95% CI**	***P*** **value**	**Heterogeneity (I** ^**∧**^ **2)**	***P*** **value of** **heterogeneity**
SCLC	0.24	[0.18; 0.30]	NS *p* = 0.425	96.56%	<0.001 <0.001
NSCLC	0.27	[0.17; 0.39]		98.57%	
male vs. female	1.15	[0.86; 1.53]	NS *p* = 0.354	75.40%	<0.001
<60 years vs. ≥60 years	0.97	[0.80; 1.19]	NS *p* = 0.773	0.00%	0.657
ECOG ≤ 1 vs. ECOG>1	0.80	[0.59; 1.09]	NS *p* = 0.161	70.10%	0.001
LD vs. ED stage	1.17	[0.60; 2.28]	NS *p* = 0.655	87.8%	<0.001
**Overall survival rates**					
**Hyponatremic vs. Normonatremic patients:**					
10 months	0.59	[0.47; 0.74]	<0.001	81.1%	<0.001
20 months	0.44	[0.33; 0.59]	<0.001	40.5%	0.109
**Hyponatremic SCLC vs. NSCLC patients at 10 months:**					
SCLC	0.42	[0.27; 0.57]	<0.001	92.72%	<0.001
NSCLC	0.27	[0.12; 0.44]	<0.001	-	-
**Corrected vs. Uncorrected hyponatremic patients:**					
10 months	1.83	[1.37; 2.44]	<0.001	0.0%	0.775
20 months	2.65	[0.94; 7.50]	0.067	23.3%	0.271

*ES, effect size; RR, risk ratio; CI, confidence interval; SCLC, small cell lung cancer; NSCLC, non-small cell lung cancer; ECOG, Eastern Cooperative Oncology Group performance status; LD, limited disease; ED, extensive disease*.

**Figure 2:**
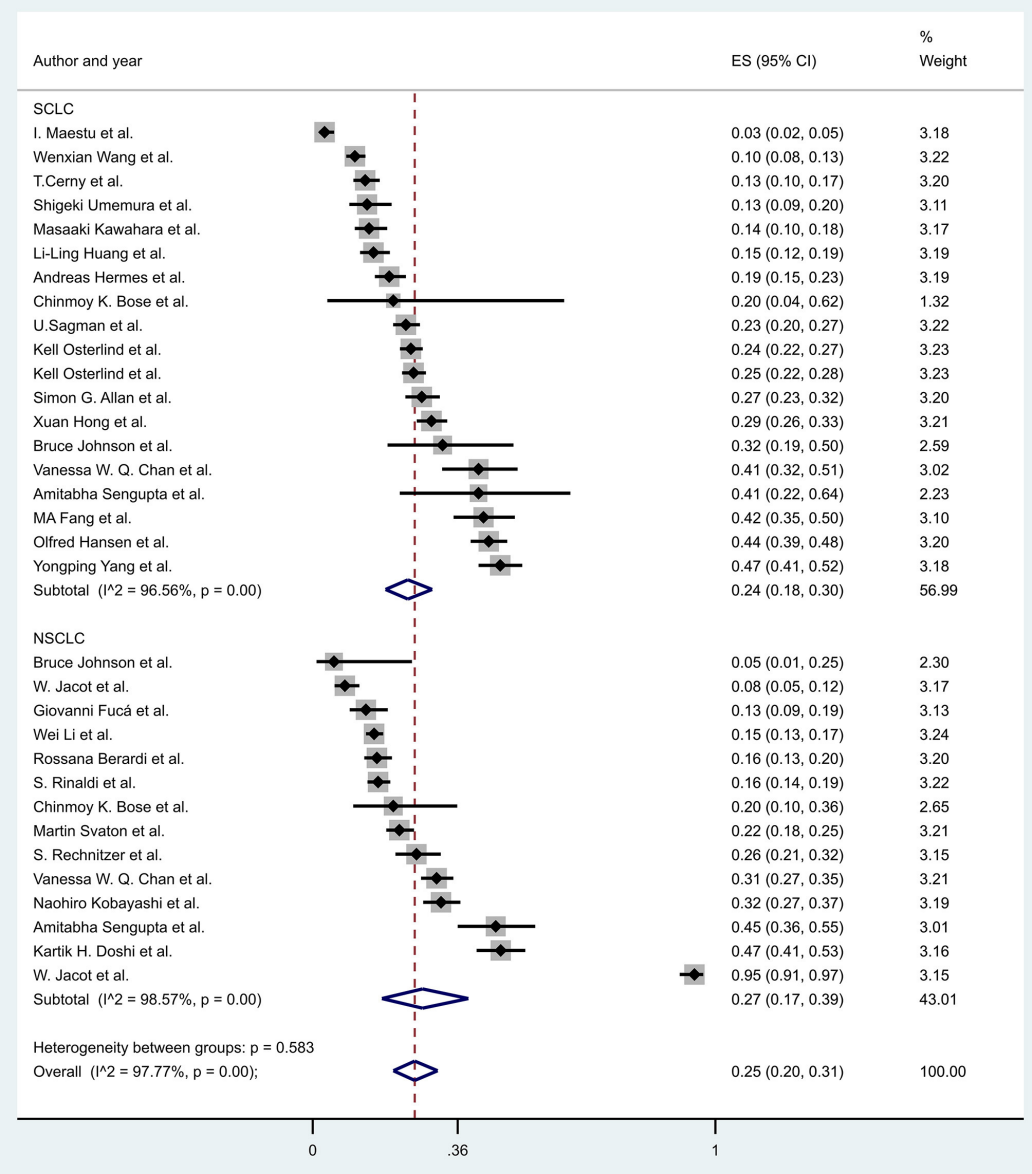
The prevalence of hyponatremia in patients with SCLC and NSCLC. Black diamonds represent the effect and vertical lines of the individual studies show the corresponding 95% CI. The size of the gray squares reflects the weight of a particular study. The blue diamond is the overall or summary effect. The outer edges of the diamonds represent the CIs. SCLC, small cell lung cancer; NSCLC, non-small cell lung cancer; ES, effect size.

**Figure 3:**
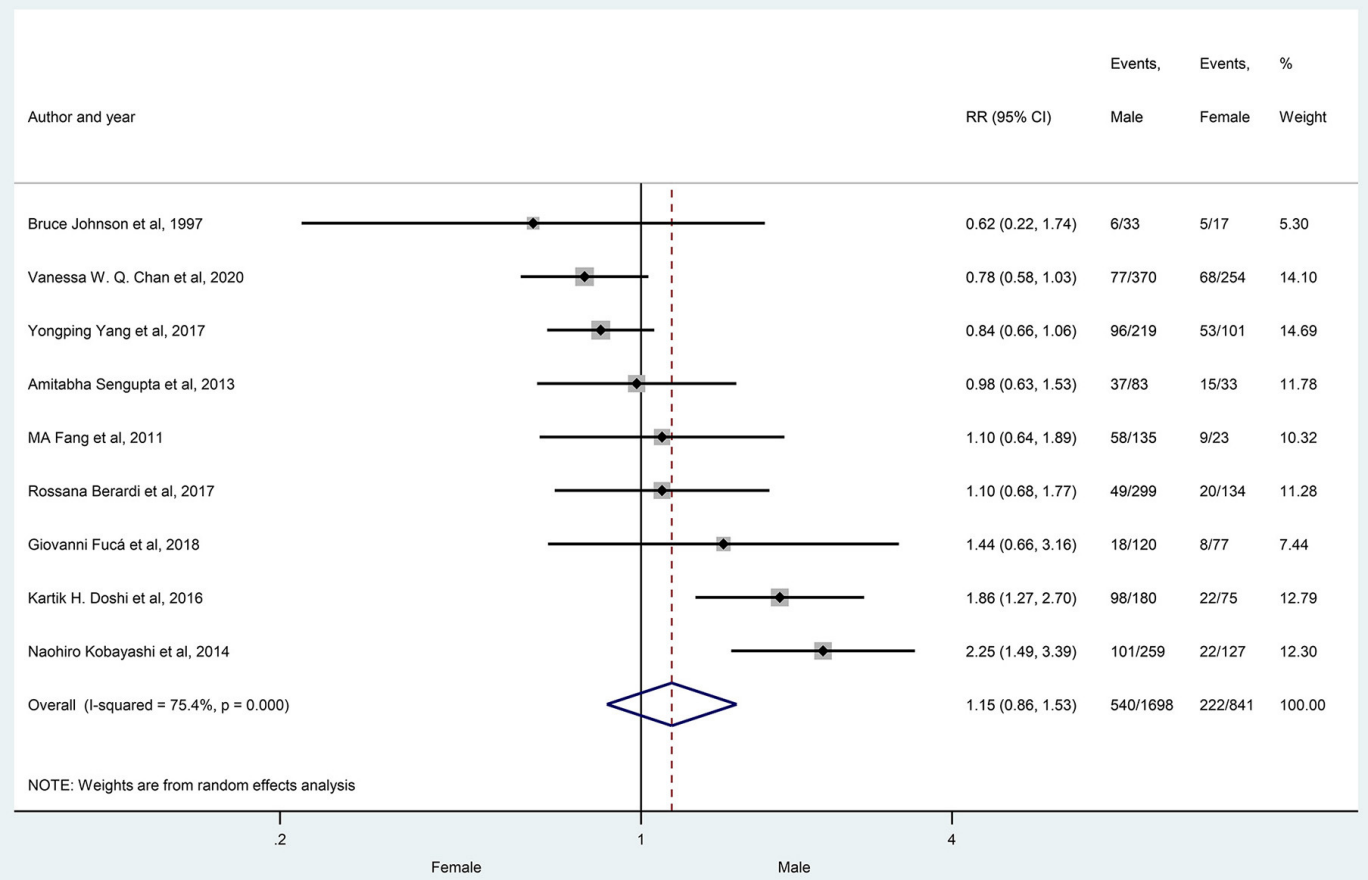
The prevalence of hyponatremia in male and female patients with lung cancer. Black diamonds represent the individual effects of studies, and vertical lines show the corresponding 95% CI. The size of the gray squares reflects the individual weight of a particular study. The blue diamond shows the overall or summary effect. The outer edges of the diamonds represent the CIs. RR, risk ratio.

**Figure 4:**
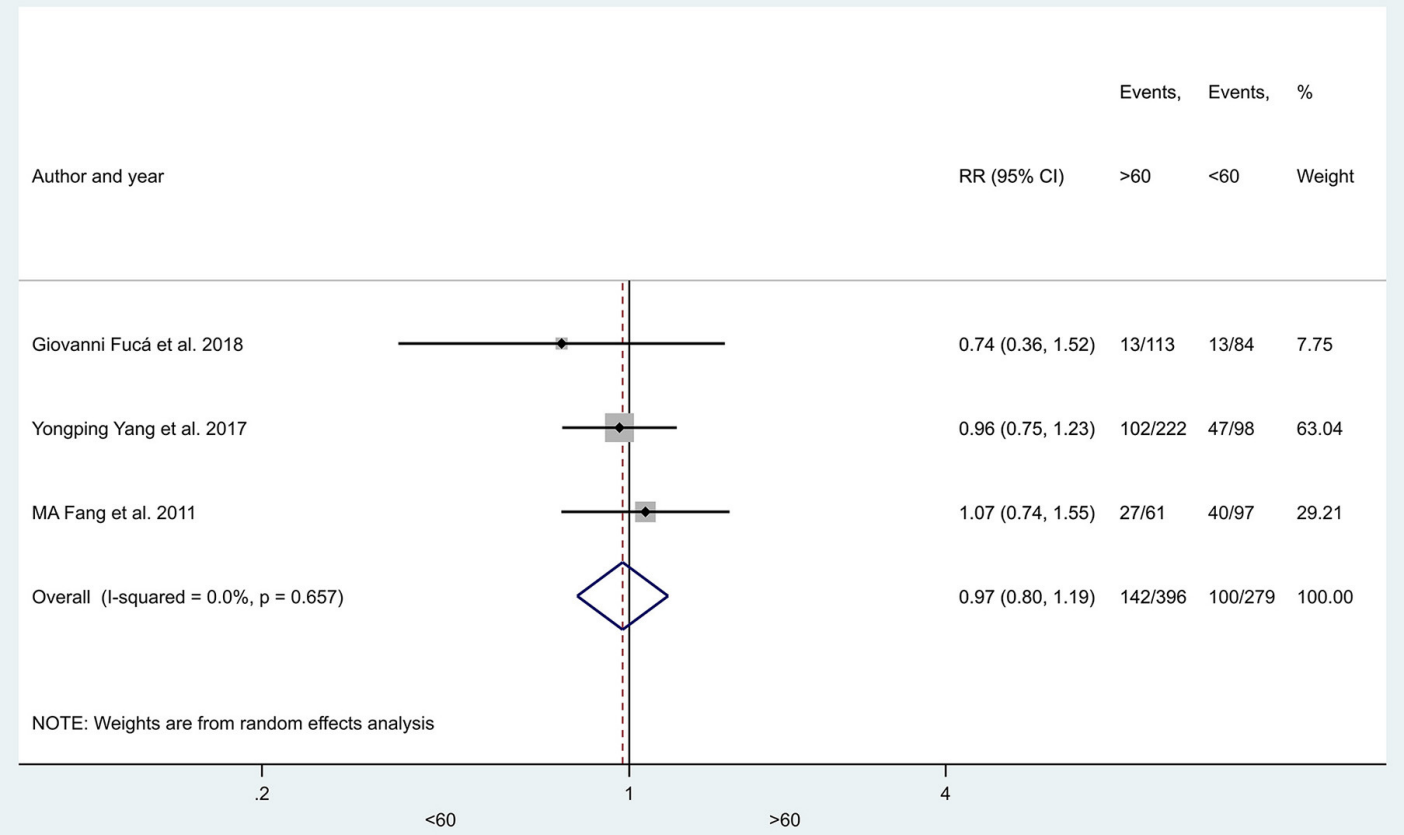
The prevalence of hyponatremia in young (<60 years) and older patients (> 60 years) with lung cancer. Black diamonds represent the individual effects of studies, and vertical lines show the corresponding 95% CI. The size of the gray squares reflects the individual weight of a particular study. The blue diamond shows the overall or summary effect. The outer edges of the diamonds represent the CIs. RR, risk ratio.

**Figure 5:**
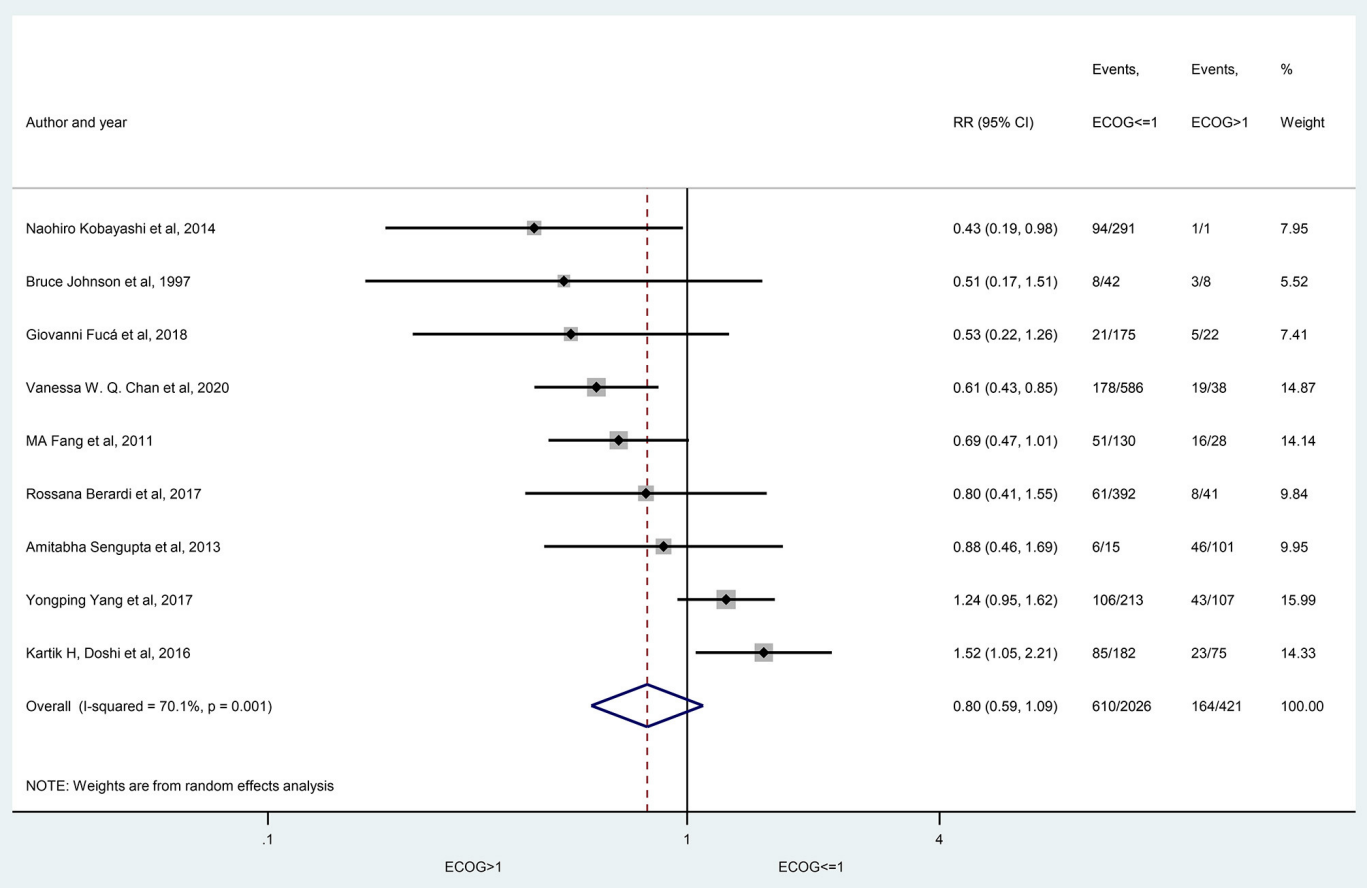
The prevalence of hyponatremia in patients with lung cancer having ECOG ≤ 1 and ECOG>1 performance state. Black diamonds represent the individual effects of studies, and vertical lines show the corresponding 95% CI. The size of the gray squares reflects the individual weight of a particular study. The blue diamond shows the overall or summary effect. The outer edges of the diamonds represent the CIs. ECOG, Eastern Cooperative Oncology Group performance status, RR, risk ratio.

**Figure 6:**
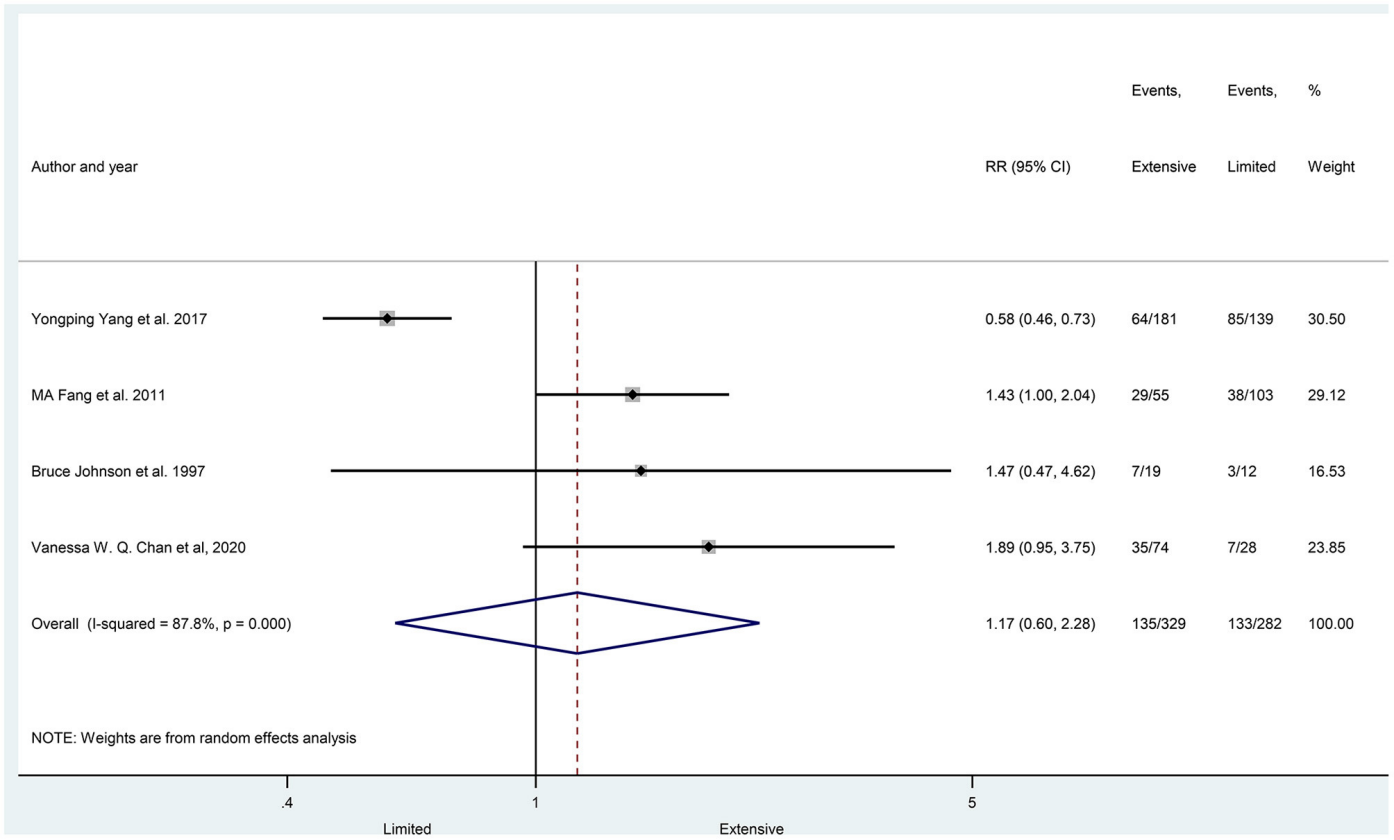
The prevalence of hyponatremia in patients with SCLC having limited and extensive disease stage. Black diamonds represent the individual effects of studies, and vertical lines show the corresponding 95% CI. The size of the gray squares reflects the individual weight of a particular study. The blue diamond shows the overall or summary effect. The outer edges of the diamonds represent the Cis. SCLC, small cell lung cancer, RR, risk ratio.

The prevalence of hyponatremia in patients with lung cancer varied between 3 and 94.8% (3–46.6% in SCLC and 5.3–94.8% in NSCLC) with an average of 25% (24% SCLC and 27% NSCLC) in the evaluated reports without significant differences between patients with SCLC vs. patients with NSCLC (29 articles, *p* = 0.425) (Figure 2), or in the subgroups created by the following criteria: gender (nine articles, *p* = 0.354), age (three articles, *p* = 0.773), ECOG state (nine articles, *p* = 0.161), the extent of disease (four articles, *p* = 0.655) (Figures 3–6).

The meta-analysis of the overall survival rates of lung cancer patients with hyponatremia was performed on eight selected studies (median follow-up time, 40 months), and a subgroup analysis was also carried out, comparing patients with SCLC and NSCLC.

**Figure 7:**
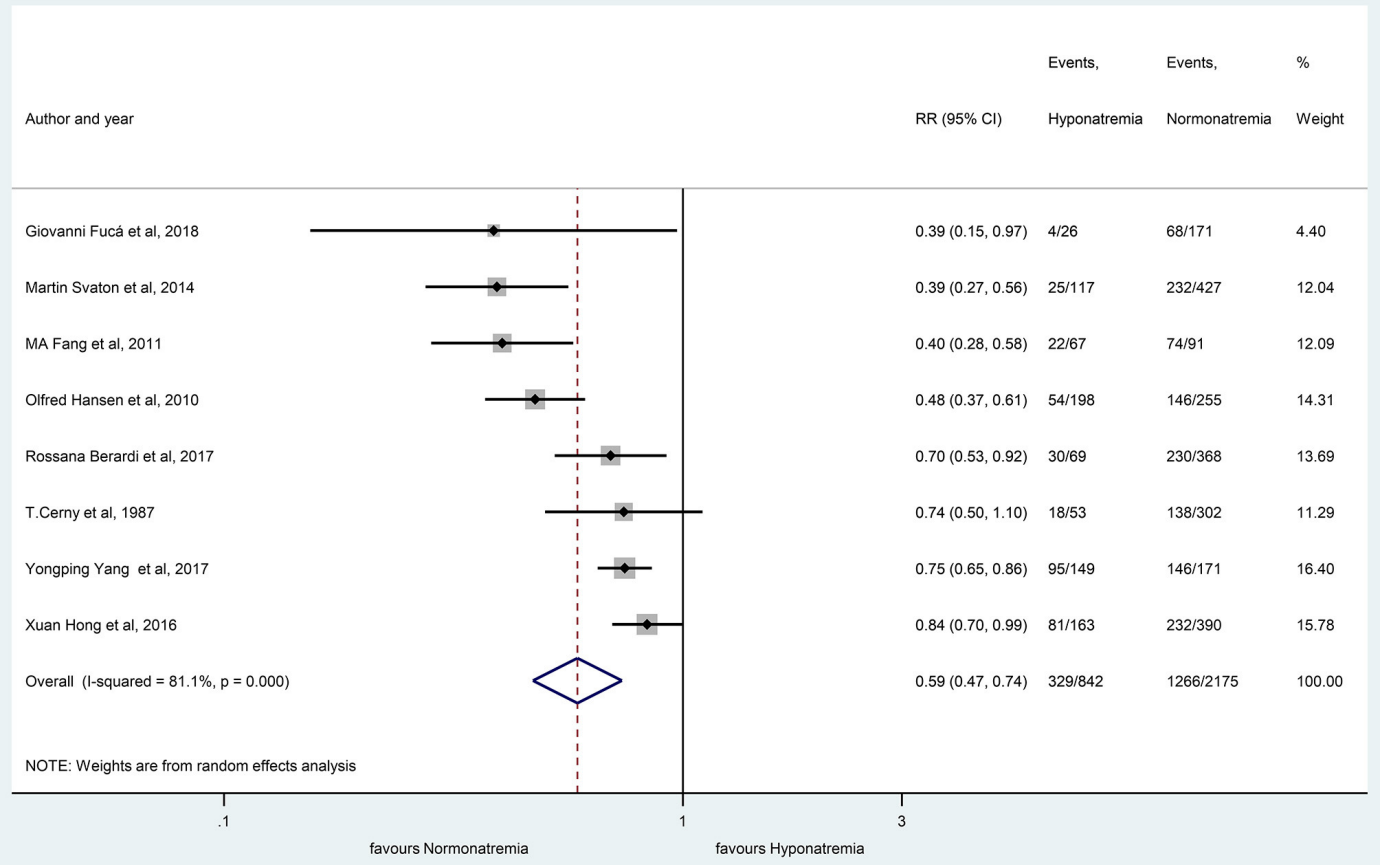
The overall survival rates at 10 months, comparing patients with hyponatremic and normonatremic lung cancer. Black diamonds represent the individual effects of studies, and vertical lines show the corresponding 95% CI. The size of the gray squares reflects the individual weight of a particular study. The blue diamond shows the overall or summary effect. The outer edges of the diamonds represent the CIs. RR, risk ratio.

**Figure 8:**
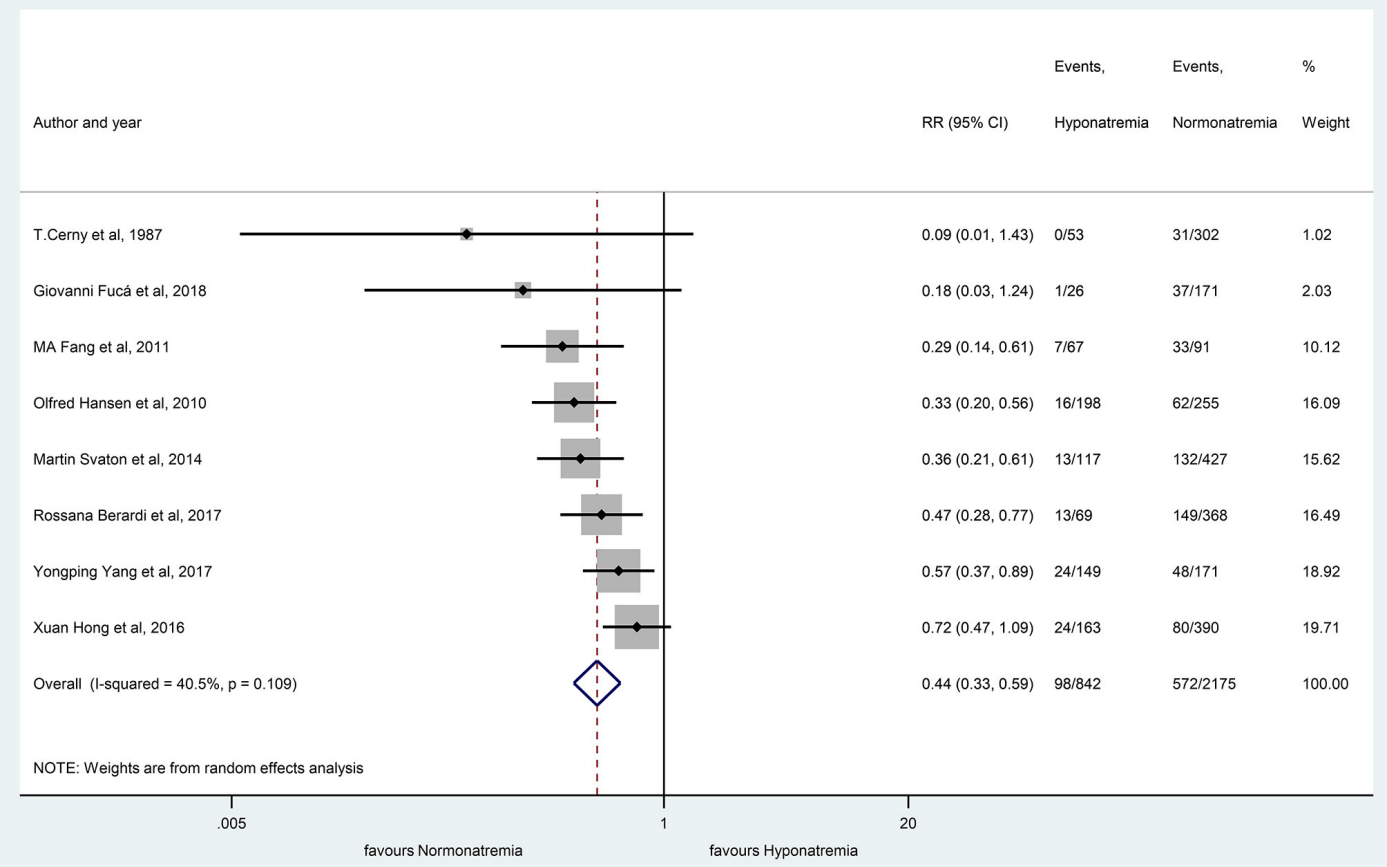
The overall survival rates at 20 months, comparing patients with hyponatremic and normonatremic lung cancer. Black diamonds represent the individual effects of studies, and vertical lines show the corresponding 95% CI. The size of the gray squares reflects the individual weight of a particular study. The blue diamond shows the overall or summary effect. The outer edges of the diamonds represent the CIs. RR, risk ratio.

The patients with hyponatremia, in comparison to patients with normonatremia, had significantly lower OS at 10 months (RR, 0.59 with 95% CI:0.47–0.74, *p* < 0.001) and 20 months (RR, 0.44 with 95% CI:0.33–0.59, *p* < 0.001), respectively, as shown in Figures 7, 8.

**Figure 9:**
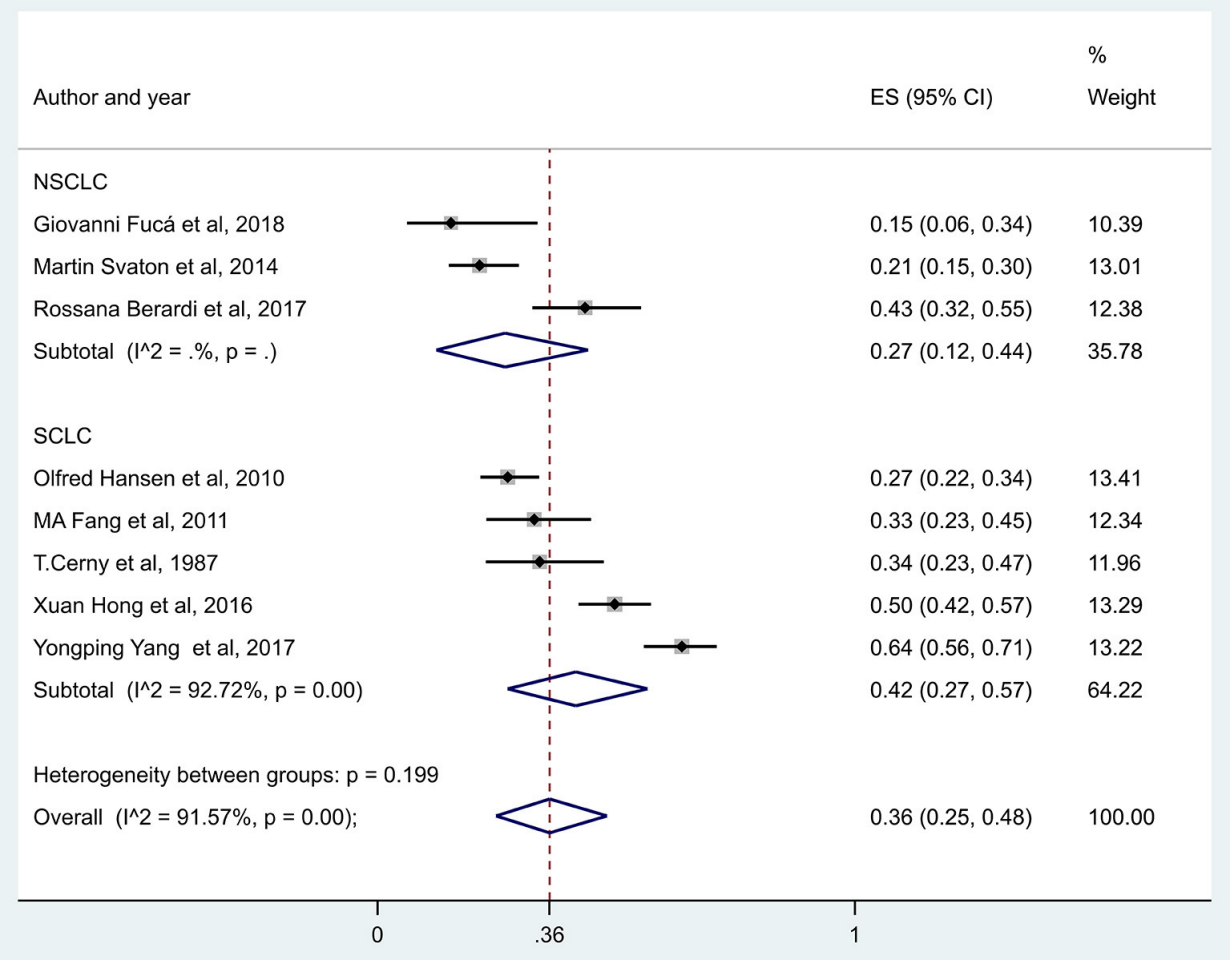
The overall survival rates at 10 months in patients with hyponatremia, comparing patients with small cell and non-small cell lung cancer. Black diamonds represent the individual effects of studies, and vertical lines show the corresponding 95% CI. The size of the gray squares reflects the individual weight of a particular study. The blue diamond shows the overall or summary effect. The outer edges of the diamonds represent the CIs. SCLC, small cell lung cancer, NSCLC, non-small cell lung cancer, RR, risk ratio.

You can see in Figure 9 that there was a lower overall survival rate at 10 months in patients with hyponatremic NSCLC (RR, 0.27, with 95% CI:0.12–0.44, *p* < 0.001) compared to patients with hyponatremic SCLC (RR, 0.42, with 95% CI:0.27–0.57, *p* < 0.001). However, no direct statistical comparison was performed.

**Figure 10:**
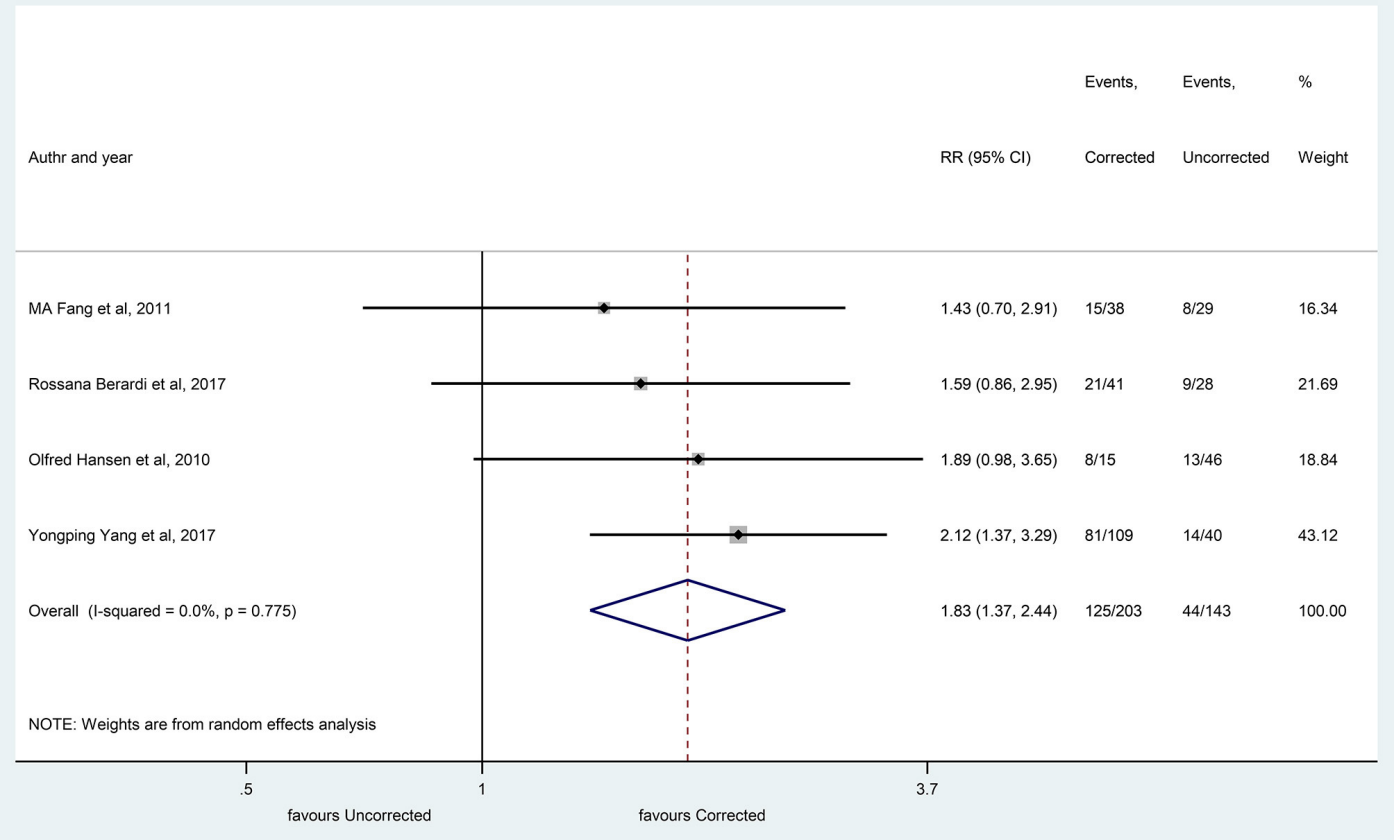
The overall survival rates after correction of hyponatremia at 10 months (corrected and uncorrected hyponatremic patient groups). Black diamonds represent the individual effects of studies, and vertical lines show the corresponding 95% CI. The size of the gray squares reflects the individual weight of a particular study. The blue diamond shows the overall or summary effect. The outer edges of the diamonds represent the CIs. RR, risk ratio.

Information about the improvement of overall survival rates, following the correction of hyponatremia, was available in only four studies. In the selected articles, the changes in serum sodium levels were the result of oncologic, symptomatic, or supportive treatment, including at least two cycles of chemotherapy, sodium supplementation, or fluid restriction. After the correction of hyponatremia, the overall survival rates at 10 months were significantly higher when compared with the uncorrected hyponatremia group (RR, 1.83 with 95% CI: 1.37–2.44, *p* < 0.001) (Figure 10).

**Figure 11:**
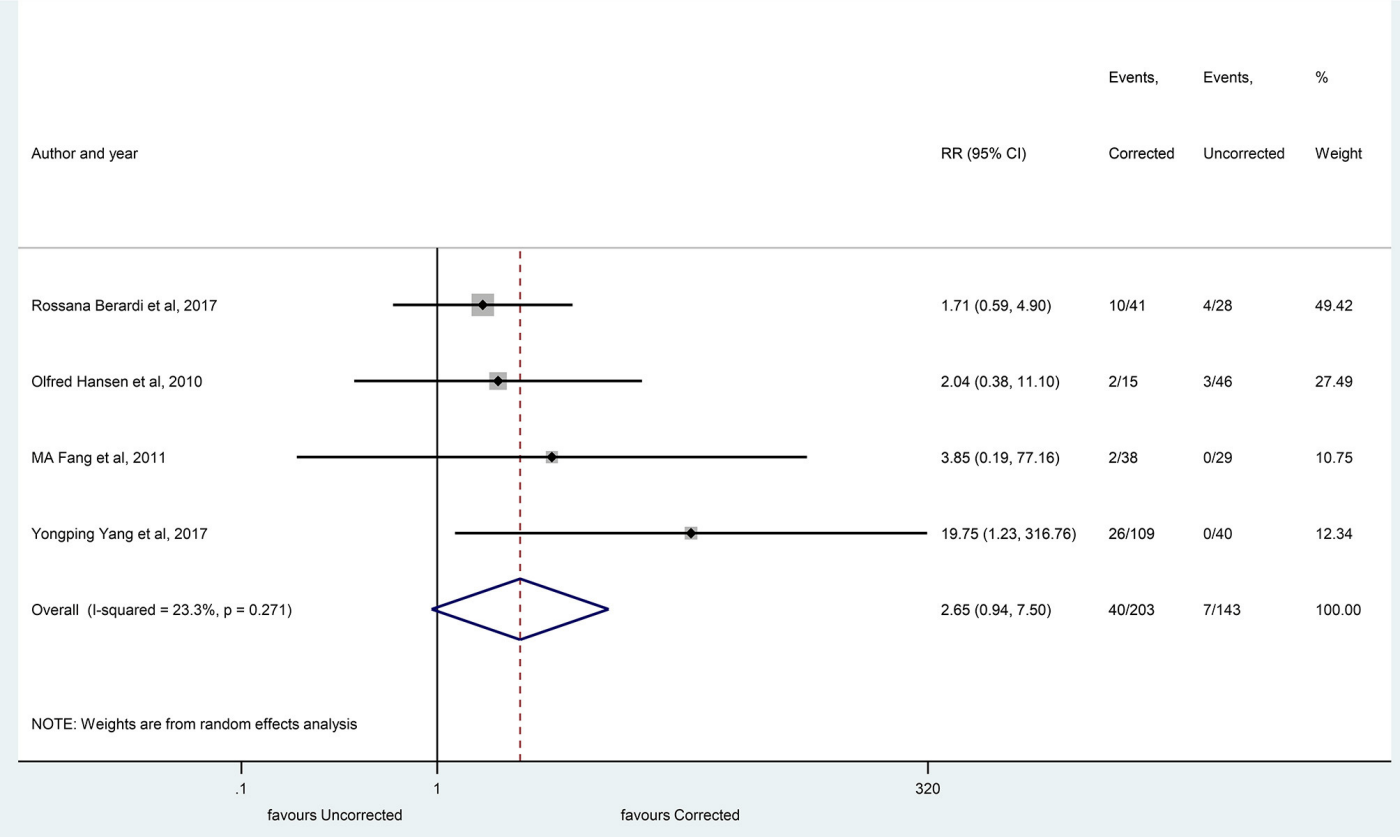
The overall survival rates after correction of hyponatremia at 20 months (corrected and uncorrected hyponatremic patient groups). Black diamonds represent the individual effects of studies, and vertical lines show the corresponding 95% CI. The size of the gray squares reflects the individual weight of a particular study. The blue diamond shows the overall or summary effect. The outer edges of the diamonds represent the CIs. RR, risk ratio.

However, at 20 months, no significant differences were identified between these groups (RR, 2.65 with 95% CI:0.95–7.50, *p* = 0.067), as shown in Figure 11.

## Discussion

Regarding the prevalence of hyponatremia in patients with lung cancer, discordant results have been published. In 1 review, the incidence of hyponatremia was suggested to be in the range of 15–75% among patients with lung cancer (3). At the same time, in another one, the prevalence varied in a lower range, from 5 to 36%, with an average of 15% in SCLC, and 4–2% in NSCLC (6). In other reviews, the prevalence of hyponatremia and/or SIADH was even less: Grohe et al. found hyponatremia in 14% of patients with SCLC and 2.7% of NSCLC (7), while Christoforos Efthymiou et al. showed that SIADH was present in 7–16% of patients with SCLC and was rare among patients with NSCLC (1%) (8).

To the best of our knowledge, ours is the first article which is not only a systematic review but also a meta-analysis evaluating the prevalence of hyponatremia in patients with lung cancer. According to our systematic search for the studies available in the literature, the prevalence of hyponatremia varies between 3. and 94.8%, with a pooled prevalence of 25% in patients with lung cancer. However, contrary to the general concept, our comprehensive evaluation and statistical analyses showed no significant difference in the prevalence of hyponatremia between SCLC and NSCLC (24 vs. 27% pooled mean frequency). The prevalence of hyponatremia has not differed significantly in the other subgroups either. So, in contrast to reviews (58, 59) and studies about the general hospitalized populations (60, 61), in patients with lung cancer, the presence of hyponatremia is not associated with age or gender. In respect of ECOG performance status or disease extent, ours is the first meta-analysis to describe that the prevalence of hyponatremia is independent of these parameters in lung cancer. Furthermore, ethnicity seems to be an important factor as well. In the article of K. Doshi and colleagues, ethnicity has been reported to be a risk factor in hyponatremia, but, unfortunately, we did not have enough data to analyze this aspect; therefore, more ethnicity-focused studies are needed (42).

The differences in the reported frequency values of low serum sodium levels in the cited reviews may be caused by the lack of appropriate studies; different eligibility criteria (hyponatremia at admission or during hospitalization); various patient groups (general hospitalized, general cancer, or only patients with SCLC/NSCLC and other subgroups); and different cut-off values (130–136 mmol/L).

Hyponatremia has an overall negative impact on mortality (5, 62–64). In a meta-analysis carried out in various hyponatremic patient groups, a significant association was revealed between hyponatremia and overall mortality (20). However, the 1,47,948 patients with hyponatremia included (from 81 studies) were not only subjects with different solid tumors but also patients with other medical conditions, such as myocardial infarction, heart failure, liver cirrhosis, and pulmonary infections. In a prospective study of patients with cancer, the mortality was higher among the patients with hyponatremic cancer compared to the whole cancer population (19.5 vs. 6.3%) (5). In a systematic review analyzing the prognostic role of hyponatremia in malignancies, including lung cancer, it was found to be a negative prognostic factor (19). In lung cancer, many authors also identified hyponatremia as an independent negative risk factor (30, 32, 34, 35). At the same time, Umemura and colleagues found that vasopressin (AVP) is a better prognostic factor than sodium levels (51). In another systematic review, the causes, diagnosis, management, and the role of hyponatremia were evaluated among patients with lung cancer. They concluded that hyponatremia is a negative prognostic and predictive factor. Furthermore, they highlighted the importance of assessing the serum sodium levels of patients with lung cancer to improve their quality of life (12). We could confirm that hyponatremia is significantly associated with worse overall survival rates in patients with lung cancer.

To the best of our knowledge, this is the first meta-analysis comparing the prognostic significance of pretreatment hyponatremia in patients with NSCLC and SCLC.

According to our analysis, hyponatremia has a more significant negative impact on the overall mortality of patients with NSCLC compared to patients with SCLC, since the overall survival rates at 10 months were significantly lower in patients with hyponatremic NSCLC than in hyponatremic SCLC ones (*p* < 0.001).

We found only one systematic review in the literature analyzing the independent prognostic role of hyponatremia in lung cancer (19). The authors identified hyponatremia as an independent risk factor in a poor outcome in 6 out of 13 and in 1 out of 3 studies in SCLC and NSCLC, respectively. However, this review has some limitations. In the included studies, hyponatremia was detected both before and during treatment, the overall survival times and rates were only partially available, and the multivariate analysis of hyponatremia was reported only in a few studies (19). In our systematic review, we came to similar conclusions regarding patients with SCLC: overall, out of the 12 studies, six identified hyponatremia as an independent prognostic factor. However, in NSCLC, we found low serum sodium to be a significant independent negative predictor more consistently (*N* = 10 vs. only one paper where it was not).

We also found another meta-analysis similar to ours after updating our systematic search. In contrast to our article, this meta-analysis evaluated the prognostic significance of hyponatremia in patients with NSCLC only, and it did not analyze the prevalence of hyponatremia and its effect on OS rates. In this systematic research, some low-quality studies with a too small number of data on prevalence and prognostic significance were also included; therefore, we chose not to include these articles in our evaluation. Regardless of the differences, their conclusion was similar to ours. They found that hyponatremia is a frequent electrolyte abnormality among patients with NSCLC, and it increases mortality, more pronounced if it remains uncorrected (65).

The improvement of hyponatremia correlated with mortality in retrospective studies in various medical conditions, including lung cancer (32, 66, 67). However, in some reviews, it was suggested that the impact of hyponatremia correction on mortality should be further investigated because it still seems to be unclear (68–70). In a meta-analysis, including 15 studies with 13,816 patients with hyponatremia, the correction of serum sodium level was achieved in 53.2% of cases, and it was associated with a significant reduction in overall mortality (23). Similarly, we found significantly higher OS rates at 10 months among patients with lung cancer if the hyponatremia was ameliorated compared to the uncorrected hyponatremia group. However, when assessed later, at 20 months, this benefit of the treatment was no longer statistically significant, which is in contrast with the observation of the previous meta-analysis carried out in various medical conditions, where reduced mortality could be detected even at 36 months. This discrepancy between the 10- and 20-month prognostic data may be related to the higher progression rate of lung cancer and, therefore, a shorter beneficial effect of the correction of the sodium level compared to other (malignant) diseases. However, statistical uncertainties (e.g., discrepant ratios of data available and/or loss of patients to follow-up during the time interval) may also influence this observation. In the meta-analysis of Cantini and colleagues, the risk of electrolyte disorders was evaluated among patients with lung cancer treated with immune checkpoint inhibitors (ICIs). Their conclusion was similar to ours; however, they investigated the incidence of treatment-related hyponatremia among patients with lung cancer. They stated that electrolyte disorders should be monitored frequently and corrected promptly because hyponatremia is associated with a worse prognosis (71).

These results show the importance of the correction of hyponatremia and raise awareness about further therapeutic options in treatment-resistant cases. Numerous papers highlighted the beneficial effect of V2R antagonists (mostly Tolvaptan), with close monitoring of sodium levels in the treatment of patients with hyponatremic lung cancer (72–78). These studies reported effective correction of hyponatremia and improvement of performance status, without any serious adverse events. However, according to our systematic search, in patients with lung cancer, the effect of V2R antagonist treatment on mortality has only been evaluated in one study, which also demonstrated the cost-effectiveness of tolvaptan treatment in Swedish patients with SIADH in pneumonia or SCLC (79). In a review of Fiordoliva et al., they found that SIADH is frequently associated with SCLC, but it should be expected in other lung cancer histotypes as well (12). We plan to investigate the diagnostic and therapeutic approaches of hyponatremia caused by SIADH in patients with lung cancer in the future.

### Limitations

The most significant limitations of our meta-analysis are the following: the lack of randomized controlled trials, the low number of prospective studies, the limited number of studies available in respect of certain relevant questions, the considerable heterogeneity of the included studies, and the variable cut-off values for serum sodium levels, which all can lead to biased results. The heterogeneity among methods of measuring serum sodium is also a limiting factor. The severity of hyponatremia at the time of diagnosis could potentially have been influenced by some interfering factors, which were not fully evaluated, such as comorbidities and treatments. Also, the lack of randomization between uncorrected and corrected hyponatremia may lead to bias.

From the available data, no certain answer can be given to the question of whether the negative prognostic significance of resistant hyponatremia was due to oncological unresponsiveness or inappropriate sodium correction. Furthermore, incomplete data reporting of follow-up time and loss of the patients to follow-up, as well as the demographic characteristics of the patients with hyponatremia have also been important limitations, e.g., lack of information about demographic characteristics of patients with hyponatremia may distort the results. More data for a better understanding would be desirable.

## Conclusions

In the literature, the prevalence of hyponatremia in patients with lung cancer varies between 3 and 94.8% with a pooled mean frequency of 25%. In our meta-analysis, no significant differences could be observed in the prevalence of hyponatremia among various subgroups, including SCLC vs. NSCLC.

Hyponatremia significantly and negatively influences the overall survival rates of these patients, especially in NSCLC where data are more consistent. Moreover, the improvement of serum sodium levels by specific or symptomatic treatment may improve the survival rates, at least in the short term.

Overall, the contradictions in the literature suggest that better-designed studies are necessary to assess the prevalence and prognostic significance of hyponatremia.

## Data Availability Statement

The original contributions presented in the study are included in the article/Supplementary Material, further inquiries can be directed to the corresponding author.

## Author Contributions

MG, EB, BT, SK, MF, PH, ZS, EM, and LB: conception and design. EB and MS: administrative support. PH, EM, and LB: provision of study materials or patients. EB and MG: collection and assembly of data. EB, MG, and DN: data analysis and interpretation. MS: manuscript writing. MS: manuscript writing. All authors: funding acquisition. All authors read and approved the final manuscript.

## Funding

This study was funded by GINOP-2.3.2-15-2016-00048 - STAY ALIVE co-financed by the European Union (European Regional Development Fund) within the framework of Programme Széchenyi 2020 and by the Human Resources Development Operational Programme Grant, Grant Number: EFOP 3.6.2-16-2017-00006 – LIVE LONGER, which is co-financed by the European Union (European Regional Development Fund) within the framework of Programme Széchenyi 2020.

## Conflict of Interest

The authors declare that the research was conducted in the absence of any commercial or financial relationships that could be construed as a potential conflict of interest.

## Publisher's Note

All claims expressed in this article are solely those of the authors and do not necessarily represent those of their affiliated organizations, or those of the publisher, the editors and the reviewers. Any product that may be evaluated in this article, or claim that may be made by its manufacturer, is not guaranteed or endorsed by the publisher.

## Supplementary Material

Supplementary File 1 A preferred reporting items for systematic reviews and meta-analyses (PRISMA) checklist.

Supplementary File 2 A Quality in Prognostic Studies (QUIPS) tool for risk of bias assessment.
