# Supplementary material for: Prevalence and Prognostic Significance of Hyponatremia in Patients With Lung Cancer: Systematic Review and Meta-Analysis
Source: Front Med (Lausanne). 2021 Dec 7;8:671951. doi: 10.3389/fmed.2021.671951 (PMC8688712; doi:10.3389/fmed.2021.671951)
Supplement: Supplementary File 2 — A Quality in Prognostic Studies (QUIPS) tool for risk of bias assessment. [file Data_Sheet_2.PDF]

Quality in Prognostic Studies (QUIPS) tool for Risk of Bias Assessment

Study participation:

- The study sample adequately represents the population of interest
- Adequate participation in the study by eligible persons
- Description of the source population or population of interest
- Description of the baseline study sample
- Adequate description of the sampling frame and recruitment
- Adequate description of the period and place of recruitment
- Adequate description of inclusion and exclusion criteria

**High Risk**	The relationship between the PF and outcome is very likely to be different for participants and eligible nonparticipants
**Moderate Risk**	The relationship between the PF and outcome may be different for participants and eligible nonparticipants
**Low Risk**	The relationship between the PF and outcome is unlikely to be different for participants and eligible nonparticipants

Study Attrition:

- The study data available (i.e., participants not lost to follow-up) adequately represent the study sample
- Adequate response rate for study participants
- Description of attempts to collect information on participants who dropped out
- Reasons for loss to follow-up are provided
- Adequate description of participants lost to follow-up
- There are no important differences between participants who completed the study and those who did not

**High Risk**	The relationship between the PF and outcome is very likely to be different for completing and noncompleting participants
**Moderate Risk**	The relationship between the PF and outcome may be different for completing and noncompleting participants
**Low Risk**	The relationship between the PF and outcome is unlikely to be different for completing and noncompleting participants

Prognostic Factor Measurement:

- The PF is measured in a similar way for all participants
- A clear definition or description of the PF is provided
- Method of PF measurement is adequately valid and reliable
- Continuous variables are reported or appropriate cut points are used
- The method and setting of measurement of PF is the same for all study participants
- Adequate proportion of the study sample has complete data for the PF
- Appropriate methods of imputation are used for missing PF data

**High Risk**	The measurement of the PF is very likely to be different for different levels of the outcome of interest
**Moderate Risk**	The measurement of the PF may be different for different levels of the outcome of interest

**Low Risk**	The measurement of the PF is unlikely to be different for different levels of the outcome of interest

Outcome Measurement:

- The outcome of interest is measured in a similar way for all participants
- A clear definition of the outcome is provided
- Method of outcome measurement used is adequately valid and reliable
- The method and setting of outcome measurement is the same for all study participants

**High Risk**	The measurement of the outcome is very likely to be different related to the baseline level of the PF
**Moderate Risk**	The measurement of the outcome may be different related to the baseline level of the PF
**Low Risk**	The measurement of the outcome is unlikely to be different related to the baseline level of the PF

Study Confounding:

- Important potential confounding factors are appropriately accounted for
- All important confounders are measured
- Clear definitions of the important confounders measured are provided
- Measurement of all important confounders is adequately valid and reliable
- The method and setting of confounding measurement are the same for all study participants
- Appropriate methods are used if imputation is used for missing confounder data
- Important potential confounders are accounted for in the study design
- Important potential confounders are accounted for in the analysis

**High Risk**	The observed effect of the PF on the outcome is very likely to be distorted by another factor related to PF and outcome
**Moderate Risk**	The observed effect of the PF on outcome may be distorted by another factor related to PF and outcome
**Low Risk**	The observed effect of the PF on outcome is unlikely to be distorted by another factor related to PF and outcome

Statistical Analysis and Reporting:

- The statistical analysis is appropriate, and all primary outcomes are reported
- Sufficient presentation of data to assess the adequacy of the analytic strategy
- Strategy for model building is appropriate and is based on a conceptual framework or model
- The selected statistical model is adequate for the design of the study
- There is no selective reporting of results

**High Risk**	The reported results are very likely to be spurious or biased related to analysis or reporting
**Moderate Risk**	The reported results may be spurious or biased related to analysis or reporting
**Low Risk**	The reported results are unlikely to be spurious or biased related to analysis or reporting
